# Open resource metagenomics: a model for sharing metagenomic libraries

**DOI:** 10.4056/sigs.1974654

**Published:** 2011-11-23

**Authors:** J.D. Neufeld, K. Engel, J. Cheng, G. Moreno-Hagelsieb, D.R. Rose, T.C. Charles

**Affiliations:** 1 University of Waterloo, Department of Biology, Waterloo, ON, Canada; 2 Wilfrid Laurier University, Department of Biology, Waterloo, ON, Canada

**Keywords:** sharing, metagenomic libraries, functional complementation

## Abstract

Both sequence-based and activity-based exploitation of environmental DNA have provided unprecedented access to the genomic content of cultivated and uncultivated microorganisms. Although researchers deposit microbial strains in culture collections and DNA sequences in databases, activity-based metagenomic studies typically only publish sequences from the hits retrieved from specific screens. Physical metagenomic libraries, conceptually similar to entire sequence datasets, are usually not straightforward to obtain by interested parties subsequent to publication. In order to facilitate unrestricted distribution of metagenomic libraries, we propose the adoption of *open resource metagenomics*, in line with the trend towards open access publishing, and similar to culture- and mutant-strain collections that have been the backbone of traditional microbiology and microbial genetics. The concept of *open resource metagenomics* includes preparation of physical DNA libraries, preferably in versatile vectors that facilitate screening in a diversity of host organisms, and pooling of clones so that single aliquots containing complete libraries can be easily distributed upon request. Database deposition of associated metadata and sequence data for each library provides researchers with information to select the most appropriate libraries for further research projects. As a starting point, we have established the Canadian MetaMicroBiome Library (CM^2^BL [1]). The CM^2^BL is a publicly accessible collection of cosmid libraries containing environmental DNA from soils collected from across Canada, spanning multiple biomes. The libraries were constructed such that the cloned DNA can be easily transferred to Gateway® compliant vectors, facilitating functional screening in virtually any surrogate microbial host for which there are available plasmid vectors. The libraries, which we are placing in the public domain, will be distributed upon request without restriction to members of both the academic research community and industry. This article invites the scientific community to adopt this philosophy of *open resource metagenomics* to extend the utility of functional metagenomics beyond initial publication, circumventing the need to start from scratch with each new research project.

## Introduction

Microbial communities harbor the immense genetic diversity that controls Earth’s biogeochemical cycling. This genetic diversity has a concomitantly immense potential for applications in bio-product synthesis, green chemistry and pharmaceutical and bio-energy sectors. Metagenomic libraries provide a window into this largely untapped reservoir of nucleic acid diversity. Individual metagenomic libraries have been generated from a variety of terrestrial and aquatic environments, and these have been prepared either as sequence-based libraries for analysis and submission to public databases, or as DNA libraries captured in a host organism (usually *Escherichia coli*) and stored as a collection of clones.

Sequencing technologies have progressed over the past decade to the point where the size of new sequence-based metagenomic and metatranscriptomic datasets often eclipses the sum of all previous database collections. Although the complete assembly of genomes from DNA sequence data generated from most environmental samples is still usually foiled by the immense genetic diversity in most microbial communities, the annotation and comparison of metagenomic sequence data can reveal functional trends that help explain adaptations of microbes to their respective habitats. Environmental sampling and sequence collection have been made possible in even modestly funded research laboratories by the advent of post-Sanger sequence platforms. Given the deluge of sequence data, the Genomic Standards Consortium (GSC) has recognized the drawbacks of the lack of sample metadata submission, or the submission of metadata structured at the discretion of individual researchers [2,3]; the recent publication of minimum information about any (x) sequence (MIxS) specifications represents a current example of essential metadata standards for adoption by the scientific community [4]. These standards will provide order and consistency as DNA sequence analysis of microbial communities continues to expand.

While the scientific community recognizes the importance of appropriate metadata collection for genomics and metagenomics research, the accompanying physical metagenomic libraries are not commonly generated and maintained as shared resources. Since the publication of the first metagenomic libraries from marine water samples [5,6], nucleic acids from diverse terrestrial, aquatic and host-associated environments have been captured in plasmid, fosmid, cosmid or bacterial artificial chromosome (BAC) libraries. The construction of these libraries requires considerable effort on the part of highly skilled bench scientists. The ends of these cloned fragments are often sequenced but, importantly, the libraries themselves are subjected to functional screening or selection for specific genes and functions. However, nearly every functional metagenomics study follows a similar methodological approach: researcher collects samples, constructs a metagenomic library, retrieves clones of immediate interest for further analysis, and stores the library until publication or a later time point. For each new study, the collection and repetition of each step could be rendered unnecessary if appropriate previously constructed libraries were available.

### Physical metagenomic libraries

Given that the success of a phenotypic screen or selection hinges on the screening strategy, expression host and the function being sought, the libraries are able to yield nearly limitless value for additional combinations and comparisons in later studies. Several examples already exist of individual laboratories mining the same metagenomic libraries for myriad functions across multiple studies. One of the earliest metagenomic studies involved capturing soil DNA in large-insert BAC libraries, followed by screens to identify clones that coded for activities including DNase, antibacterial, lipase, amylase, cellulase, chitinase, esterase, keratinase and protease [7]. The same soil library (SL2) was then used for (a) screening and recovery of genes coding for the production of turbomycin A and B [8], (b) linking ribosomal RNA genes with additional genetic material [9] and (c) identifying clones conferring antibiotic resistance [10]. All of these SL2 screens were done in *E. coli* as the host. Another example of multiple applications for metagenomic libraries includes cosmid libraries generated from activated sludge and soils. These originally underwent selection for clones conferring the ability to utilize D-3-hydroxybutyrate as sole carbon source in both *E. coli* and *Ensifer meliloti* (*Sinorhizobium meliloti*) surrogate hosts [11]. Subsequently, several of these libraries were screened to retrieve luxR-luxI type quorum sensing systems [12], phosphate metabolism genes [13], and poly-3-hydroxybutyrate synthesis genes [14], all in *E. meliloti* or *Agrobacterium tumefaciens* as a surrogate host. These examples illustrate the sustained value of metagenomic libraries for use in a range of hosts for uncovering a wide variety of different genes, enzymes and functions related to small-molecule metabolites in the environment.

### Sharing model in science

Unlike the examples above, most metagenomic libraries are prepared for individual projects and are not necessarily maintained in a manner that facilitates convenient and low-cost distribution. This situation hinders both repetition of the work done previously and exploration of the captured DNA for additional enzymatic functions. These libraries, similar to cultured isolates or mutant strains, could have value extending far beyond the initial publication. According to the *International Journal of Systematic and Evolutionary Microbiology*, “characterization of prokaryote strains must include all relevant metadata (e.g. location isolated, strain designations and culture collections, and other environmental variables)” [15]. For mutant strains, the *Molecular Microbiology* journal stipulates that “authors will distribute freely any strains, clones or antibodies described therein for use in academic research”, which is similar to the recommendation for authors from the *Journal of Bacteriology* that mutant strains be “available from a national collection or will be made available in a timely fashion, at reasonable cost, and in limited quantities to members of the scientific community for noncommercial purposes”.

A tradition of sharing has been integral to the development of the science of microbiology. From the earliest days, sharing of pure cultures has been essential to experimental replication and validation, and for the definition of bacterial types. The validity of patent claims requires access to cultures, and such access is legally mandated. Central culture collections such as the American Type Culture Collection (ATCC), Deutsche Sammlung von Mikroorganismen und Zellkulturen (DSMZ) and hundreds of others around the world [16] have ensured strain availability and the scientific community has rallied to ensure strain maintenance [17]. These conventions do not yet have equivalents for metagenomic libraries.

One reason for the lack of storage and distribution standards for physical metagenomic libraries may be that the customary formats for storage are 96- or 384-well plates. Multi-well plates require extensive storage space. For example, a single 100,000-clone metagenomic library from a soil sample would be represented by approximately ~1000 96-well plates or ~250 384-well plates, which could fill an entire ultrafreezer with only 96-well plates. Due to the obvious challenges of permanent storage of large numbers of plate-arrayed metagenomic libraries, we argue that libraries should also be preserved in a format that is amenable to low-cost shipping and storage so these valuable resources remain available for long-term distribution subsequent to publication.

### Open resource metagenomics

We propose an open resource model for archiving published libraries using an alternative clone pooling strategy for storage and distribution. The features of this model include (a) cloning of large-size inserts into versatile vectors for downstream screening or selection in a variety of hosts, (b) pooling of clones for facilitating convenient library distribution in standard microcentrifuge tubes, and (c) extensive metadata and associated sequence-based characterization of libraries.

Cloning of inserts into broad host-range vectors helps circumvent one of the main limitations of metagenomic library functional screens: the inability to express many genes in a single host (e.g. *E. coli*). Indeed, screening in multiple hosts can reveal target clones that would not have been evident otherwise [11]. Because successful expression of heterologous genes is dependent on the surrogate host employed, it is desirable to construct libraries in vectors compatible with a variety of host organisms. Given the specific host-range requirements of different library vectors, it is unlikely that a universal vector, able to replicate in all possible hosts, will be developed. Instead, one approach is to employ Gateway® technology [18] in cosmid library vectors to enable *en masse* transfer of cloned genomic DNA from the library vector backbone to host-specific destination vectors, resulting in expression clones appropriate for the desired surrogate host.

Our recommended protocol for pooling clones is less labor intensive than picking colonies for distribution into multi-well plates. Instead of growth and storage of arrays of clones in individual wells, we suggest that libraries initially grown as colonies be sequentially washed from agar surfaces with fresh liquid culture medium. Alternatively, selection of clones during library construction can be carried out directly in liquid culture as demonstrated previously [19]. The resulting volume of medium with a dense collection of clones can be distributed into frozen aliquots for future dilution and plating for screens, or transfer to defined media for targeted selection protocols. These colony aliquots or DNA preparations can be shipped to collaborating laboratories for further investigation (e.g. the SL2 library has been distributed in this way; Jo Handelsman, personal communication).

To facilitate direct access to these libraries by members of the worldwide scientific community, sample information can be retrieved from a selection of database fields such as the environmental context, the preparation method, and a link to a sequence annotation server such as MG-RAST [20] where associated sequence data, and sequence-based phylogenetic and metabolic information can be hosted. The cost of sequencing has decreased such that bulk DNA and 16S rRNA gene sequences can be obtained affordably to provide large-scale phylogenetic and metabolic characterization of each environmental sample. These sequence data represent an important complement to detailed physical, chemical and geographic information that comprise the MIxS specifications. Internally, the Handlebar database [21] can allocate unique barcodes for samples and libraries.

Altogether, these approaches are affordable and would result in libraries being made readily available to all researchers, and allow future grant proposals to leverage existing libraries from international research colleagues. Each functional study using the library could be related to the sequence data, as well as other functional studies using the same library, and would ensure that the original creators of the library are appropriately cited. Open resource metagenomics helps circumvent the traditional cycle of requiring new samples to be collected for each subsequent study. With such an approach, metagenomic libraries could be considered akin to isolates and mutant strains that are housed in culture collections. Unlike established culture collections, our recommendation would be that open resource metagenomics be established conceptually as a federated system in which individual laboratories maintain control and maintenance of pooled metagenomic libraries, ready for distribution upon request. As the *ethos* of open resource metagenomics and demand for access to libraries builds, it is not inconceivable that central repositories (e.g. ATCC, DSMZ) could be approached to offer permanent storage for these genetic resources. Ultimately, the scientific value of environmental metagenomic libraries is analogous to the agricultural value of seeds from Earth’s plants, which are protected within a distributed system of Seedbanks.

Of course, as with isolates and mutant strains, there remain outstanding challenges that could prevent universal adoption of open resource metagenomics. For example, many funding agencies require and encourage the involvement of for-profit industry, which typically includes agreements with regards to intellectual property. Under these circumstances, the reality may be that unique arrangements will be required to blend open resource metagenomics with industrial collaborations. These arrangements may include delayed library release or the maintenance of particular libraries outside of the research literature. In addition, some laboratories may require a specific security level for labs handling and receiving metagenomic material and others may require signed agreements for transfers of research materials. Of course another important barrier to a central repository would be financial implications. As with culture collections, sustained funding is essential to maintain resources for sample submission, cataloguing and distribution. Although we are seeking appropriate funding towards establishing such a centralized system, the methodological approaches outlined above will enable the immediate adoption of a distributed open resource system for researchers preparing new metagenomic libraries. These are issues that we are working to resolve and we urge the research community to join this effort in moving toward a system of open resource metagenomics for greatest international collaborative benefit.

### Starting point initiative

**Figure 1:**
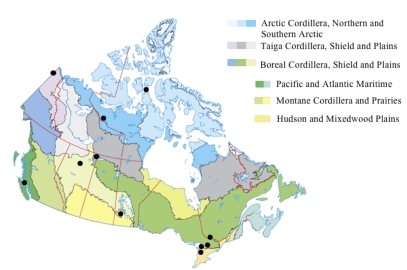
Terrestrial ecozones of Canada. The location of initial soil samples for the Canadian MetaMicroBiome Library is indicated with a black circle (•). The map was modified from the Canadian Soil Information System.

In order to demonstrate that such an open resource approach is feasible, we have initiated the Canadian MetaMicroBiome Library project (CM^2^BL [1]), a publicly accessible collection of libraries of environmental DNA representing Canadian soil microbial communities. Canada spans a variety of natural regions, or ecozones (Figure 1). Its 20 ecozones consist of 15 terrestrial and 5 marine regions. The CM^2^BL was initiated with the construction of cosmid libraries cloning DNA isolated from soil samples collected from across Canada spanning multiple biomes and ecozones (Figure 1). These samples are being characterized by high-throughput DNA sequencing to determine the taxonomic, genetic, and metabolic diversity of each sample alongside screens for industrially relevant enzymes. Once the proof-of-principle stage of CM^2^BL is complete, contributions of samples from other environments will be considered for inclusion, provided additional samples are sufficiently distinct to maximize captured microbial diversity. The resulting resources will provide the international scientific community with access to a large collection of thoroughly characterized genetic materials in clone library format for phenotypic screening or selection for enzymes with truly novel functions, independent from sequence-based surveys. We invite other scientists who are regularly generating libraries to make them publicly available in a similar manner, and to work with us to develop an exchange system so that libraries are mirrored in multiple archive locations.

The CM^2^BL libraries are prepared using derivatives of the IncP cosmid pRK7813 [22] that have been converted to Gateway® entry vectors. This allows the insert DNA to be easily transferred to Gateway® vectors of diverse host range using either *in vivo* [23] or *in vitro* [24] reactions, thus facilitating phenotypic screening of the libraries in bacterial and yeast hosts of interest. We store backups at various stages of library development: DNA extracts, ligation products, packaged phage particles, clone libraries in *E. coli* and as extracted cosmid clone DNA. For each sample prepared thus far, libraries contain pools of between 10,000 and 250,000 cosmids. Copies of these libraries are stored as permanent frozen archives and purified cosmid DNA, in duplicate freezers with CO_2_ backup systems to ensure security of the resource. If required, additional library expansions can be generated for distribution to companies or academic institutions at minimal cost per library. Distribution of libraries is accompanied by materials transfer agreements to ensure consistency with requirements of the UN Convention on Biodiversity [25].

**Figure 2:**
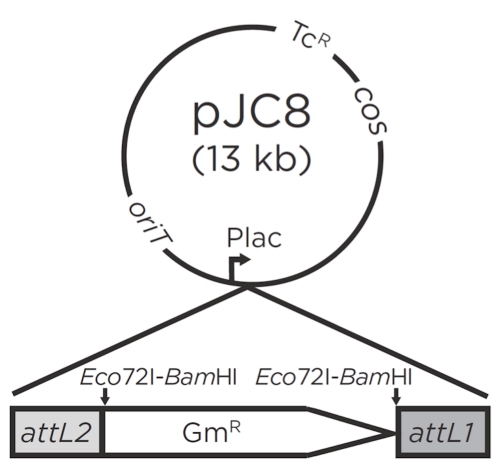
Gateway® entry cosmid pJC8. *Bam*HI and *Eco*72I sites are used for cloning sticky and blunt ends of inserts, respectively. The *cos* site is used for *in vitro* packaging of the recombinant cosmid DNA into bacteriophage λ heads; *oriT* (RK2 origin of transfer) site is used for the transfer of cosmid clones from *E. coli* to other bacterial hosts.

The CM^2^BL resource was initiated by preparing a cornfield soil metagenomic library using the versatile IncP cosmid vector pJC8 (pRK7813 derivative; Figure 2), allowing for phenotypic screening in a broad range of microbial surrogate hosts. Cosmid pJC8 is a low-copy and broad-host-range cosmid. The cosmid library was constructed using Stratagene packaging extracts and *E. coli* HB101 (3.2 × 10^5^ clones formed/µg insert DNA). A total of 79,058 clones with ~33 kb random inserts have been generated. The resulting soil DNA library was calculated to contain 2,640 Mb of metagenomic DNA, which represents approximately 561 genomes, assuming average bacterial genome size of 4.7 Mb in the soil sample [26].

## Conclusion

Considering that methods for preparing metagenomic libraries have largely remained constant since their conception in the 1990s, these proposed standards for metadata, storage and distribution of metagenomic libraries are anticipated to remain relevant for decades. We are expanding CM^2^BL to include samples from additional environments and have partnered with international research initiatives such as the Earth Microbiome Project to provide sequence-based context for libraries intended for functional screens and selections. Ultimately, open resource metagenomics, and initiatives such as CM^2^BL, will provide the international scientific community with cost-recovery access to a collection of thoroughly characterized genetic materials for the phenotypic screening of enzymes and other gene products with truly novel functions independent of sequence-based surveys, available for distribution upon request.
